# Equilibrium properties of assembly of interacting superparamagnetic nanoparticles

**DOI:** 10.1038/s41598-020-70711-w

**Published:** 2020-08-13

**Authors:** N. A. Usov, O. N. Serebryakova

**Affiliations:** 1 National University of Science and Technology “MISiS”, Moscow, 119049 Russia; 2 Pushkov Institute of Terrestrial Magnetism, Ionosphere and Radio Wave Propagation, Russian Academy of Sciences, IZMIRAN, Troitsk, Moscow, 108480 Russia

**Keywords:** Magnetic properties and materials, Materials science, Nanoscience and technology

## Abstract

The stochastic Landau–Lifshitz equation is used to investigate the relaxation process and equilibrium magnetization of interacting assembly of superparamagnetic nanoparticles (SPMNPs) uniformly distributed in a nonmagnetic matrix. For weakly interacting assembly, the equilibrium magnetization is shown to deviate significantly from the Langevin law at moderate and large magnetic fields under the influence of their magnetic anisotropies. For dense assemblies with noticeable influence of the magneto-dipole interaction, a significant dependence of the initial susceptibility on the assembly density is revealed. The difference between the initial susceptibility and the corresponding Langevin susceptibility can serve as an indication of appreciable influence of the magneto-dipole interaction on the assembly properties. A new self-consistent approach is developed to explain the effect of mutual magneto-dipole interaction on the behavior of dense assembly of SPMNPs. The probability densities of the components of random magnetic field acting on magnetic NPs are calculated at thermodynamic equilibrium. The self-consistent probability densities of these components are found to be close to Gaussian distribution. A decreasing equilibrium assembly magnetization as a function of its density can be explained as a disorienting effect of the random magnetic field on the NPs magnetic moments.

## Introduction

Assemblies of superparamagnetic nanoparticles (SPMNPs) are widely used in various fields of nanotechnology, in particular, in biomedicine, for magnetic resonance imaging, targeted drug delivery, purification of biological media from toxins, in magnetic hyperthermia, etc.^1–4^. However, the study of the physical properties of dense assemblies of magnetic NPs is complicated by the influence of a strong magneto-dipole interaction between the NPs^5–13^. Formally, the equilibrium properties of an assembly of SPMNPs distributed in a rigid media can be studied on the basis of the Gibbs principle^14–18^, if for a given temperature *T* of a thermal bath and applied magnetic field *H*_0_ the complete thermodynamic equilibrium is established for a finite time. For such assembly, the equilibrium magnetization, *M*_*eq*_ = *M*_*eq*_(*H*_0_,*T*), can be calculated as the derivative of the free energy with respect to the applied magnetic field^14–18^. Unfortunately, the direct use of the Gibbs statistical integral for calculating the equilibrium properties of dense NPs assembly is associated with great mathematical difficulties.

In a classical paper^19^, Langevin used Gibbs principle^20,21^ to calculate the equilibrium magnetization of a non-interacting assembly of freely rotating magnetic dipoles. The Langevin law for equilibrium assembly magnetization is often used in the analysis of the experimental data^22–29^. However, it is important to recall that in the simplest Langevin approximation^19^, both the magnetic anisotropy energy and the energy of the magneto-dipole interaction of NPs are neglected. Meanwhile, the influence of particle magnetic anisotropy on assembly behavior has been investigated analytically^30,31^ and numerically^32^ based on the Gibbs formula in the limit of weakly interacting NPs. On the other hand, the evaluation of the Gibbs statistical integral in a general case of interacting assembly is a difficult problem, well known in the theories of non-ideal gas, dipole fluids, plasma, and other fields of classical and quantum physics^14–18,20^. A similar problem also exists for interacting assemblies of SPMNPs.

In recent years a significant amount of research^25,33–55^ has been devoted to theoretical and experimental studies of the influence of the magneto-dipole interaction on the properties of dense NPs assemblies. In particular, the Monte-Carlo simulations have been carried out^25,33–42,46,50,53^ for assemblies of interacting magnetic NPs in the temperature range exceeding the blocking temperature. Various generalizations of the Langevin formula were proposed, such as the interacting SPM model (ISM)^43–45,51^, or different versions of the effective magnetic field^46–50,52^. For the same purpose the thermodynamic perturbation theory^54^ and the decomposition of the Gibbs statistical integral by the Born–Mayer method^55^ were employed. However, despite several approaches used, an understanding of the role of magneto-dipole interaction in equilibrium properties of dense SPMNPs assembly is still incomplete.

In this paper, the equilibrium magnetization of an assembly of interacting SPMNPs uniformly distributed in a rigid nonmagnetic matrix is calculated by solving the stochastic Landau–Lifshitz (LL) equation^56–60^. This approach is an alternative to the classical method of Gibbs assemblies. It enables one to simultaneously take into account the effect of various types of magnetic anisotropy, magneto-dipole interaction, and thermal fluctuations of the particle magnetic moments on the assembly behavior. Moreover, this method allows one to consider also kinetic processes such as the relaxation process to the equilibrium assembly magnetization.

Calculations based on the stochastic LL equation were performed in this work for a dilute assembly of NPs clusters with a finite filling density *η* = *N*_*p*_*V*/*V*_*cl*_, where *V* = π*D*^3^/6 is a volume of spherical nanoparticle of diameter *D*, *N*_*p*_ being the number of NPs in a cluster of volume *V*_*cl*_. The random positions of the NPs in the cluster are assumed to be fixed, the rotation of the NPs as a whole is excluded. The easy anisotropy axes of the NPs are randomly oriented. A saturation magnetization of the particles is taken to be *M*_*s*_ = 350 emu/cm^3^, which is typical for iron oxide NPs^3,6,10^. The uniaxial magnetic anisotropy constant *K*_1_ is varied from 6 × 10^4^ to 1.5 × 10^5^ erg/cm^3^. The numerical simulations are carried out at room temperature, *T* = 300 K. Therefore, the diameter of spherical NPs is restricted to a range *D* < 25 nm, to ensure^61^ that the blocking temperature *T*_*b*_ of the largest NPs is well below the room temperature. A significant dependence of the assembly equilibrium magnetization on the intensity of the magneto-dipole interaction inside the clusters has been revealed.

In addition, the statistical properties of random magnetic field acting on magnetic NPs in a dense assembly of SPMNPs have been studied. Following the Lorentz approach^20^ it is shown that the random component of magnetic field acting on a typical nanoparticle of the assembly is determined only by the surrounding NPs located inside the Lorentz sphere. The magnetic moment of a typical nanoparticle, and hence the equilibrium magnetization *M*_*eq*_ of the assembly, is calculated self-consistently depending on the total magnetic field acting on the particle. The variant of the self-consistent field approximation developed in this work is shown to describe qualitatively correctly the numerical simulation data for the equilibrium assembly magnetization, obtained by means of solution of the stochastic LL equation, over an effective *H*_0_ ≤ 600 Oe range.

## Results and discussion

### Dilute nanoparticle assembly

As mentioned in the introduction, there are two important contributions that lead to a difference in the reduced equilibrium magnetization of interacting assembly, *m*_*eq*_ = *M*_*eq*_(*H*_0_,*T*)/*M*_*s*_, from the Langevin law^19–21^

1 $$\frac{\left\langle M \right\rangle }{{M_{s} }} = m_{L} \left( x \right);\quad m_{L} \left( x \right) = \coth \left( x \right) - \frac{1}{x},$$

where *x* = *M*_*s*_*VH*_0_/*k*_*B*_*T* is the dimensionless Langevin variable and *k*_*B*_ is the Boltzmann constant. These are magnetic anisotropy energy and the energy of the magneto-dipole interaction. Let us discuss the influence of these factors on the equilibrium properties of an assembly separately.

**Figure 1:**
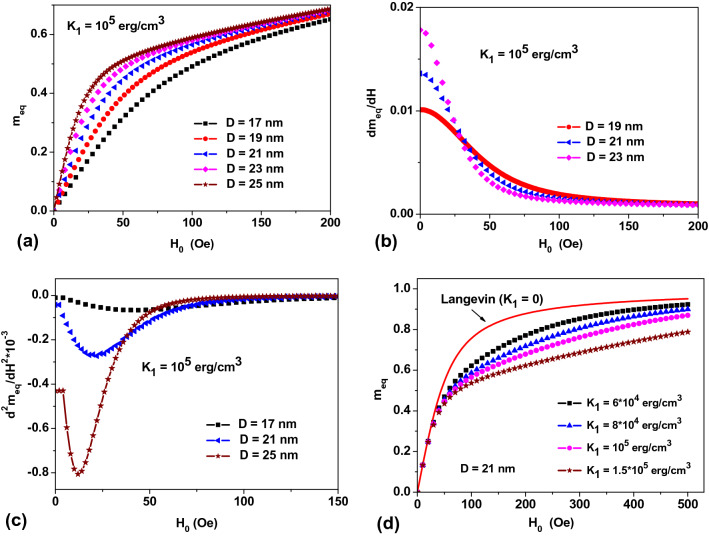
(**a**) The reduced equilibrium magnetization, *m*_*eq*_ = *M*_*eq*_/*M*_*s*_, of a randomly oriented assembly of non-interacting magnetic NPs of different average diameters; (**b**) reduced magnetic susceptibility of the assembly, *dm*_*eq*_/*dH*_0_; (**c**) a second derivative of equilibrium magnetization, showing a pronounced minimum; (**d**) a dependence of the reduced assembly magnetization on the *K*_1_ values for NPs (*D* = 21 nm) with *M*_*s*_ = 350 emu/cm^3^ at room temperature.

Consider first a relatively simple case of a dilute NPs assembly, *η* → 0, neglecting the influence of magneto-dipole interaction. Here, an equilibrium assembly magnetization can be determined evaluating the Gibbs statistical integral^30–32^. The corresponding calculations for randomly oriented monodispersed assemblies of SPMNPs are shown in Fig. 1. The magnetic field dependence of equilibrium magnetization of randomly oriented NPs of various diameters is shown in Fig. 1a. Figure 1b shows that the static magnetic susceptibility of the assembly falls down rapidly over *H*_0_ ≤ 200 Oe. As can be seen from the plots, the static susceptibility substantially depends on average *D* values. Moreover, as Fig. 1c portrays, a second derivative of *m*_*eq*_ with respect to *H*_0_ reveals a pronounced minimum in the region of low, *H*_0_ ≤ 50 Oe, magnetic fields, whose position is a function of average *D* value.

Let us normalize *H*_0_ to the particle anisotropy field, *H*_*a*_ = 2*K*_1_/*M*_*s*_, in a reduced variable *h*_*e*_ = *H*_0_/*H*_*a*_. Then it can be shown^30–32^ that a *m*_*eq*_ value in a dilute assembly is a universal function of *h*_*e*_ that depends only on the reduced height of the particle potential energy barrier, *R*_*b*_ = *K*_1_*V*/*k*_*B*_*T*, so that *m*_*eq*0_ = *m*_*eq*0_(*h*_*e*_,*R*_*b*_). However, as Fig. 1d shows in a limit of small *H*_0_ the *m*_*eq*_(*H*_0_) curve coincides with the Langevin function, Eq. (1), for all *K*_1_ values. This is a consequence of the fact that in the limit *h*_*e*_ → 0 the expansion

2 $$m_{eq0} \left( {h_{e} ,R_{b} } \right) = \frac{2}{3}R_{b} h_{e} + \cdots = \frac{{M_{s} VH_{0} }}{{3k_{B} T}} + \cdots$$

is valid^30,31^. As a result, a *K*_1_ dependence of *m*_*eq*_ in the region of small *H*_0_ disappears. At the same time, according to Fig. 1d for moderate and large *H*_0_ the difference of *m*_*eq*_ from the Langevin function is very significant. It follows from Eq. (2) that a static magnetic susceptibility of the assembly in the low-field region does not depend on the *K*_1_ value. Therefore, it is impossible to determine the *K*_1_ value by measuring the static susceptibility of a dilute assembly, *dm*_*eq*0_/*dH*_0_, in the limit *h*_*e*_ → 0. At the same time, as we will see later, the static magnetic susceptibility of an interacting assembly differs significantly from the Langevin susceptibility. This important fact makes it possible to evaluate the effect of the magneto-dipole interaction on the equilibrium properties of an assembly.

The noticeable influence of the particle magnetic anisotropy energy on the behavior of dilute assembly of monodispersed NPs in the range of moderate and high *H*_0_ fields, and at temperatures not too high with respect to *T*_*b*_ was studied in detail both experimentally^23,25^ and theoretically^30–32^. The area of parameters *H*_0_ and *T*, where there is a considerable deviation of *m*_*eq*_ from the Langevin law was characterized^23,25^ as an anisotropic superparamagnetism. Unfortunately, in a number of recent experimental works (see, for example, Refs.^26–29^), the experimental *m*_*eq*_ data are described by a weighted sum of Langevin functions. In this way, the particle size distribution is taken into account, whereas the influence of the magnetic anisotropy energy is completely ignored.

### Assembly of dense 3D clusters

As noted in the introduction, a direct application of the Gibbs principle for calculating the equilibrium magnetization of an assembly of interacting NPs is associated with significant mathematical difficulties. To overcome this difficulty, various theoretical methods were used^25,33–55^. The most convincing results were obtained by means of Monte-Carlo simulations^33–42,46,50,53^ for assemblies of interacting SPMNPs uniformly distributed in a nonmagnetic media. However, a known drawback of this method is the difficulty in estimating the actual time for evolution of the assembly in a given magnetic field, as individual Monte-Carlo steps do not correspond to real physical time^33^. As an alternative approach to the problem, in the given paper we use direct numerical simulation based on a solution of the stochastic LL equation^56–60^. Numerical calculations of *m*_*eq*_ and static susceptibility of a dilute assembly of dense clusters consisting of *N*_*p*_ = 60–100 NPs are carried out in a range of applied magnetic fields, *H*_0_ = 0–600 Oe, the cluster filling density being *η* = 0–0.3. The details of numerical modeling of the kinetic properties of an assembly of magnetic NPs using the stochastic LL equation are described below in the “Methods” section.

**Figure 2:**
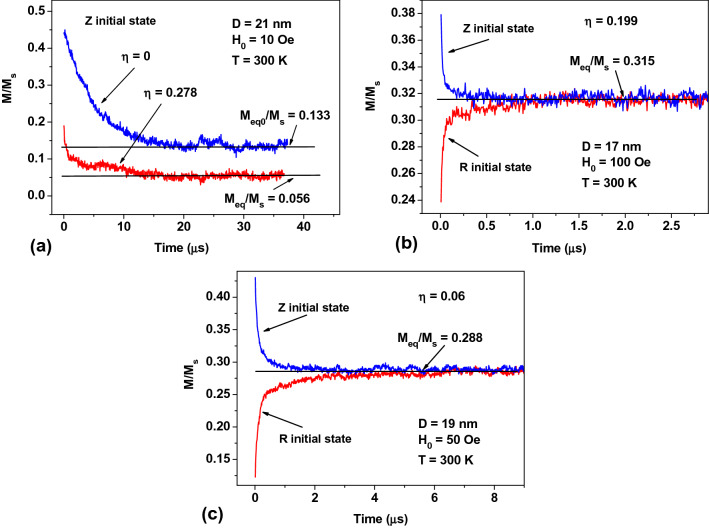
Relaxation of magnetization to a thermodynamically equilibrium value in randomly oriented assemblies of magnetic NPs: (**a**) comparison of the magnetization relaxation curves of non-interacting (*η* = 0) and interacting (*η* = 0.278) assemblies of NPs of diameter *D* = 21 nm and (**b**, **c**) comparison of magnetization relaxation curves for different initial magnetization states for NPs of *D* = 17 and 19 nm, respectively, with *M*_*s*_ = 350 emu/cm^3^ and *K*_1_ = 10^5^ erg/cm^3^.

Figure 2 shows the magnetization relaxation curves of randomly oriented assemblies of magnetic NPs of various *D* values in a given *H*_0_ field for different initial magnetization states. In the magnetization distribution designated as *Z* state, at time *t* = 0 the NPs are magnetized along the applied magnetic field, whereas for the *R* initial state the magnetic moments of the NPs are randomly oriented in space. Both initial distributions of the particle magnetic moments differ from thermal equilibrium. Figure 2 shows a temporal evolution of the assembly magnetization for *t* > 0. It is calculated by solving the stochastic LL equation with a sufficiently small numerical time step Δ*t* with respect to characteristic particle precession time *T*_*p*_^60^. To obtain the complete magnetization relaxation curve of an assembly to the equilibrium state a sufficiently large number of the numerical time steps must be taken. The thermodynamic equilibrium is considered to be achieved when the magnetic relaxation curve approaches a constant value, *m*_*eq*_ = *M*_*eq*_/*M*_*s*_, and fluctuates around this value with a small dispersion, as shown in the relaxation curves presented in Fig. 2. To obtain statistically reliable results a large number of numerical experiments, *N*_*exp*_ = 100–200, is carried out with the same initial conditions. An average magnetization of a dilute assembly of clusters is calculated by averaging over the set of magnetic relaxation curves of individual clusters with independent realization.

Figure 2a compares the magnetization relaxation curves from a uniformly magnetized state (Z state) in *H*_0_ = 10 Oe for non-interacting and interacting assemblies of NPs of the same diameter *D* = 21 nm. To obtain the statistically reliable results shown in Fig. 2a, the numerical simulation data were averaged over *N*_*exp*_ = 200 independent numerical experiments. In every numerical experiment *N* = 3 × 10^6^ numerical steps were performed with a small time step Δ*t* = 1.26 × 10^–5^ μs, assuming a phenomenological damping coefficient *κ* = 0.5. In Fig. 2a the relaxation curve for a non-interacting assembly, *η* = 0, can be described by a time dependent exponent with a single relaxation time *τ* = 0.2 μs. It approaches a steady value, *m*_*eq*0_ = 0.133, which coincides with the reduced equilibrium magnetization of the non-interacting assembly calculated using the Gibbs formula. At the same time, as Fig. 2a shows, the relaxation curve for an assembly of clusters with a filling density *η* = 0.278 cannot be characterized by a single relaxation time. To approximate this curve at least two exponents with significantly different relaxation times should be used. Nevertheless, as Fig. 2a shows, this curve also approaches a steady value, *m*_*eq*_ = 0.056, at a sufficiently long time. It is reasonable to take this value as the equilibrium magnetization of an assembly of clusters with a filling density *η* = 0.278 in applied magnetic field *H*_0_ = 10 Oe.

As Figure 2a shows, a magneto-dipole interaction leads to a decrease in the magnetization relaxation time at the fast initial stage, followed by a much slower stage of the full establishment of thermodynamic equilibrium, during which the average magnetization of the assembly already changes relatively weakly. Interestingly, the equilibrium magnetization for the assembly of clusters with a noticeable intensity of the magneto-dipole interaction always decreases compared to that of the corresponding assembly of non-interacting NPs.

This conclusion is confirmed by the data in Fig. 2b,c were the magnetization relaxation curves of various assemblies are shown for different initial *Z* and *R* states, respectively. As can be seen from Fig. 2b,c, in accordance with the Gibbs principle the equilibrium state of the assembly in a given *H*_0_ field turns out to be the same, regardless of the type of initial magnetization configuration. It is worth mentioning that the Gibbs postulate is not applicable to study a temporal evolution of the assembly magnetization. Fortunately, it can be done numerically by solving the stochastic LL equation. The equilibrium value of the reduced magnetization of the assembly can be obtained by averaging the relaxation curve over a finite interval of times exceeding the characteristic time of magnetic relaxation, *t* > *τ*.

**Figure 3:**
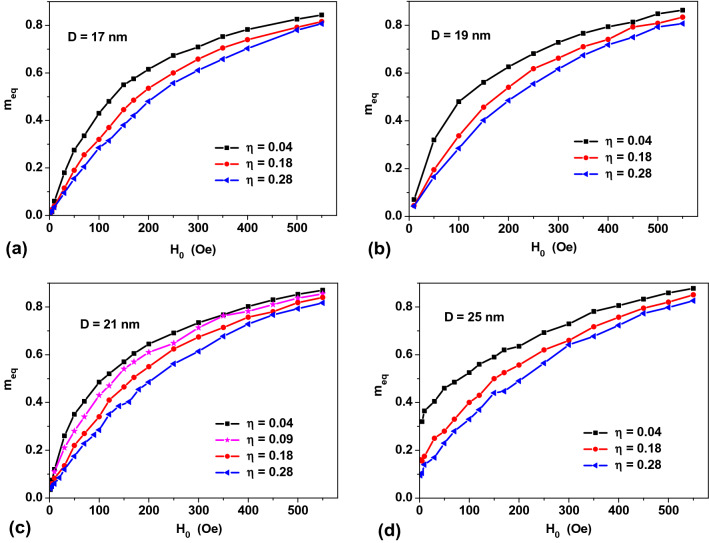
Equilibrium reduced magnetization over *H*_0_ fields for dilute assemblies of clusters of magnetic NPs with *M*_*s*_ = 350 emu/cm^3^, *K*_1_ = 10^5^ erg/cm^3^, and the number of particles in the clusters *N*_*p*_ = 60, for various *D* and *η* values at room temperature.

For an assembly with given parameters (*D*, *M*_*s*_, *K*_1_*, N*_*p*_*, η*) it is possible to obtain the equilibrium value of the reduced magnetization as a function of *H*_0_ using the calculations similar to those shown in Fig. 2b,c. The results so obtained are plotted in Fig. 3. As cluster filling density *η* increases, resulting in the increase in the magneto-dipole interaction intensity inside the clusters, the value of the equilibrium assembly magnetization decreases. In Fig. 3a–c, the magnetic susceptibility of the assembly, *dm*_*eq*_/*dH*_0_, in the low-field, *H*_0_ → 0, substantially decreases as a function of *η* values. For a given set of *M*_*s*_ and *K*_1_ values for NPs assemblies of *D* ≤ 21 nm the reduced equilibrium magnetization at room temperature vanishes in the limit *H*_0_ → 0. These assemblies exhibit typical SPM behavior. At the same time, as Fig. 3d shows, for an assembly of NPs of a larger *D* = 25 nm, there is a remanent magnetization in the limit *H*_0_ → 0. Therefore, the blocking temperature *T*_*b*_ of this assembly exceeds the room temperature value. As a result, the true equilibrium state for this assembly is not reached in a finite evolutionary time. Interestingly, in Fig. 3d the remanent magnetization of the assembly decreases with increasing intensity of the magneto-dipole interaction.

**Figure 4:**
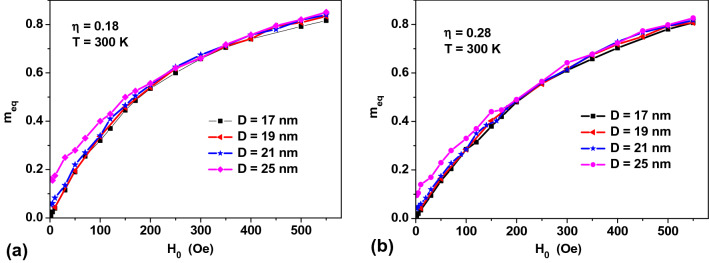
A comparison of the reduced equilibrium magnetizations for assemblies of NPs with *M*_*s*_ = 350 emu/cm^3^, *K*_1_ = 10^5^ erg/cm^3^, of different diameters, but with the same *η* values.

Figure 4 demonstrates an universal behavior of the equilibrium magnetization curves for assemblies of NPs with a noticeable intensity of the magneto-dipole interaction, *η ≥ *0.2. While the equilibrium magnetization curves of assemblies of non-interacting NPs substantially depend on an average *D* value (see Fig. 1a), these curves in interacting assemblies practically coincide at the same *η* value. An exception is the magnetization curve in rather large particles, *D* = 25 nm, with nonzero remanent magnetization.

To explain this effect, one notes that to an order of magnitude the magnetic anisotropy energy of the particle is *W*_*a*_ ~ *K*_1_*V*, whereas the characteristic energy of the magneto-dipole interaction of the NPs can be estimated as $W_{m} \sim {{\left( {M_{s} V} \right)^{2} } \mathord{\left/ {\vphantom {{\left( {M_{s} V} \right)^{2} } {L_{av}^{3} }}} \right. \kern-\nulldelimiterspace} {L_{av}^{3} }}$, where *L*_*av*_ is the average distance between the NPs of the cluster, which can be estimated from the relation $L_{av}^{3} = {{V_{cl} } \mathord{\left/ {\vphantom {{V_{cl} } {N_{p} }}} \right. \kern-\nulldelimiterspace} {N_{p} }}$. Thus, for the characteristic energy of the magneto-dipole interaction one obtains $W_{m} \sim M_{s}^{2} V\eta$. Therefore, the energy ratio ${{W_{a} } \mathord{\left/ {\vphantom {{W_{a} } {W_{m} }}} \right. \kern-\nulldelimiterspace} {W_{m} }}\sim {{K_{1} } \mathord{\left/ {\vphantom {{K_{1} } {M_{s}^{2} \eta }}} \right. \kern-\nulldelimiterspace} {M_{s}^{2} \eta }}$ is independent of the nanoparticle volume being approximately constant for a fixed *η* value.

**Figure 5:**
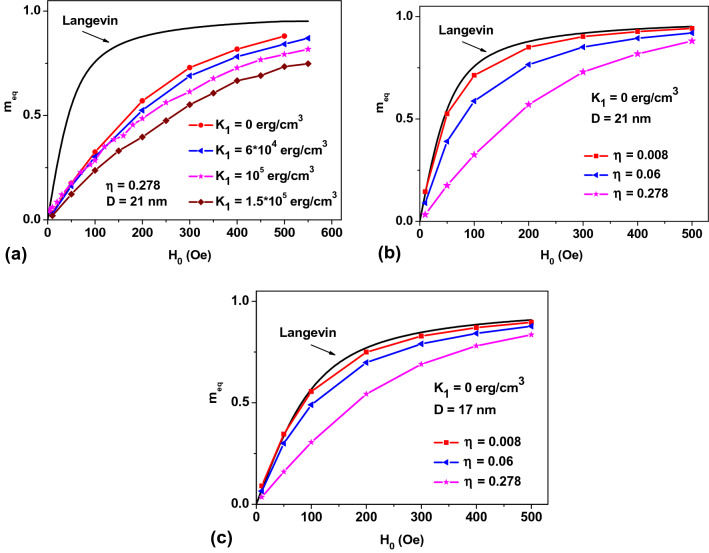
Dependence of the equilibrium reduced magnetization on: (**a**) *K*_1_ value for assemblies with a fixed *η* = 0.278 and (**b**, **c**) on *η* values for assemblies of NPs of various *D* values with *K*_1_ ~ 0.

Figure 5a plots the reduced equilibrium magnetizations of the assemblies of NPs with the same *D* = 21 nm, but with different magnetic anisotropy constants. It is noteworthy that the equilibrium magnetization of interacting NPs assemblies differs significantly from the Langevin curve. As Fig. 5a shows in a sufficiently dense assemblies with *η* = 0.278 the influence of particle magnetic anisotropy on the equilibrium magnetization curve is not significant. In particular, the static magnetic susceptibility of the assembly, *dm*_*eq*_/*dH*_0_, in the limit *H*_0_ → 0 is practically independent of the *K*_1_ value, similar to the case of assemblies of non-interacting NPs (see Fig. 1d). However, the static magnetic susceptibility of the interacting assembly is significantly less than the Langevin value, *dm*_*eq*_/*dH*_0_ = *M*_*s*_*V*/3*k*_*B*_*T*^30,31^.

To demonstrate clearly the effect of the magneto-dipole interaction on the equilibrium properties of a SPM assembly, it is of interest to study the equilibrium magnetization curves of an assembly of NPs with a negligibly small *K*_1_ ~ 0. As Fig. 5b,c show, the equilibrium magnetization in the case *K*_1_ = 0 approaches the Langevin curve only in the limit *η* → 0. Note that the magnetic susceptibility of such an assembly in the limit *H*_0_ → 0 substantially depends on its *η* value. As can be seen from Fig. 3, this conclusion is also valid for assemblies with a finite *K*_1_ value. Thus, a difference of the static magnetic susceptibility from the Langevin value, *dm*_*eq*0_/*dH*_0_ = *M*_*s*_*V*/3*k*_*B*_*T*, reveals the influence of the magneto-dipole interaction of NPs on the assembly properties.

### Self consistent field approximation

The detailed numerical calculations performed above make it possible to quantitatively assess the change in the equilibrium and kinetic properties of the assembly with an increase in the intensity of the magneto-dipole interaction. However, these calculations do not shed light on the physical cause of such changes. It is clear that in the presence of a magneto-dipole interaction, the magnetic field acting on a typical nanoparticle differs from the magnetic field *H*_0_ applied to the assembly, since the magnetic fields of the surrounding NPs also act on this nanoparticle. In dense clusters, at small distances between the NPs, the magnetic fields of the nearest NPs can be very significant. Therefore, a fundamental interest is determining a probability density of such a magnetic field acting on a typical magnetic nanoparticle in the assembly.

In recent years, several approaches are proposed^46–52^ to introduce effective magnetic field acting on a typical nanoparticle in a dense SPM assembly. However, it is shown^53^ that the expressions suggested for the effective magnetic fields in some cases are hardly consistent with the Monte-Carlo simulation results. In this paper, we develop another approach to evaluate the effect of random magnetic fields acting in a dense nanoparticle assembly.

**Figure 6:**
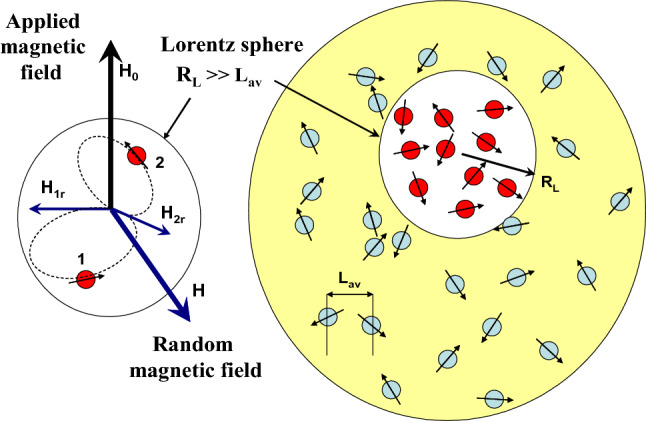
A model Lorentz sphere around a reference nanoparticle in an assembly of SPMNPs.

Let us consider an effectively large spherical assembly, as schematically shown in Fig. 6, which can be characterized by an average magnetization *M*_*eq*_(*H*_0_,*T*) at equilibrium. Let us select around a typical nanoparticle of the assembly a spherical region (Lorentz sphere^20^) with radius *R*_*L*_ much larger than the average distance *L*_*av*_ between NPs. Outside Lorentz sphere one can introduce a nearly homogeneous magnetization distribution close to the average assembly magnetization, <*M*(***r***)>  = *M*_*eq*_. Then, inside the Lorentz sphere, at least near its center, the magnetic field of external magnetic dipoles is almost completely compensated to zero^20^. Therefore, the magnetic field in the center of the Lorentz sphere acting on the reference particle is created by the surrounding NPs located in the Lorentz sphere.

First, let us analyze the probability density of random magnetic field acting on a typical particle of an assembly with a negligibly small magnetic anisotropy constant, *K*_1_ = 0. As Fig. 5b,c show, in such assembly due to the influence of the magneto-dipole interaction a difference arises between the equilibrium magnetization and the Langevin law. Let ***H*** = (*H*_*x*_, *H*_*y*_, *H*_*z*_) be a vector of the random magnetic field in the center of Lorentz sphere created by the NPs located inside it. Without a loss of generality, one can assume that the external magnetic field *H*_0_ is applied along the *Z* axis of the Cartesian coordinates. Then, the total magnetic field in the center of Lorentz sphere is given by ***H***_*t*_ = (*H*_*x*_, *H*_*y*_, *H*_*z*_ + *H*_0_). Let *H*_*t*_ be the module of this vector. It is reasonable to assume that in thermodynamic equilibrium the time-average magnetic moment of the reference particle located in the center of Lorentz sphere is

3 $$\left\langle M \right\rangle /M_{s} = m_{L} \left( {\frac{{M_{s} VH_{t} }}{{k_{B} T}}} \right),\quad H_{t} = \sqrt {H_{x}^{2} + H_{y}^{2} + \left( {H_{z} + H_{0} } \right)^{2} } ,$$

where *m*_*L*_(*x*) is the Langevin function in Eq. (1). It points parallel to vector ***H***_*t*_, so

4 $$\left\langle {M_{x} } \right\rangle /\left\langle M \right\rangle = {{H_{x} } \mathord{\left/ {\vphantom {{H_{x} } {H_{t} }}} \right. \kern-\nulldelimiterspace} {H_{t} }},\quad \left\langle {M_{y} } \right\rangle /\left\langle M \right\rangle = {{H_{y} } \mathord{\left/ {\vphantom {{H_{y} } {H_{t} }}} \right. \kern-\nulldelimiterspace} {H_{t} }},\quad \left\langle {M_{z} } \right\rangle /\left\langle M \right\rangle = {{\left( {H_{0} + H_{z} } \right)} \mathord{\left/ {\vphantom {{\left( {H_{0} + H_{z} } \right)} {H_{t} }}} \right. \kern-\nulldelimiterspace} {H_{t} }}$$

Further, let *P*(*H*_*x*_,*H*_*y*_,*H*_*z*_) be the probability density of a random magnetic field created by surrounding particles in the center of the Lorentz sphere. Then, the average magnetization of the assembly in the direction of the applied field *H*_0_ is given by

5 $$\frac{{\left\langle {M_{z} } \right\rangle }}{{M_{s} }} = \iiint {m_{L} \left( {\frac{{M_{s} VH_{t} }}{{k_{B} T}}} \right)}\frac{{H_{z} + H_{0} }}{{H_{t} }}P\left( {H_{x} ,H_{y} ,H_{z} } \right)dH_{x} dH_{y} dH_{z} .$$

Thus, to calculate the equilibrium assembly magnetization in the given approximation it is necessary to determine the probability density of random magnetic field in the center of the Lorentz sphere, created by NPs located inside the said sphere.

For given assembly parameters, a self-consistent value *P*(*H*_*x*_,*H*_*y*_,*H*_*z*_) can be obtained numerically by conducting a sufficient number of numerical experiments with random spherical clusters of a volume *V*_*cl*_, number of particles *N*_*p*_, and a fixed *η* value. As will be shown below, the partial probability densities *P*(*H*_*x*_), *P*(*H*_*y*_), and *P*(*H*_*z*_) of the random functions *H*_*x*_, *H*_*y*_, and *H*_*z*_ are close to the Gaussian distributions. Due to the random nature of the magnetic field ***H***_*t*_, which is the sum of a large number of independent contributions of the magnetic fields of individual NPs, there is a relation

6 $$P(H_{x} ,H_{y} ,H_{z} ) \, = P(H_{x} )P(H_{y} )P(H_{z} ).$$

To find self-consistent probability densities *P*(*H*_*x*_), *P*(*H*_*y*_) and *P*(*H*_*z*_), an appropriate iterative procedure should be performed. At the first stage of this procedure we consider all particles inside the Lorentz sphere to be magnetized strictly parallel to the *H*_0_ field, so that <*M*_*x*_> = 0, <*M*_*y*_> = 0, <*M*_*z*_> = *M*_*s*_. Under this assumption we obtain the empirical probability densities *P*_1_(*H*_*x*_), *P*_1_(*H*_*y*_) and *P*_1_(*H*_*z*_) of the first approximation in the following manner. A sufficiently wide range of magnetic fields, (− *H*_*max*_, *H*_*max*_), is divided into a large number of equal intervals, *ΔH* ≪ *H*_*max*_. Then a sufficient number of numerical experiments *N*_*exp*_ are performed in random clusters created independently. A random field ***H*** = (*H*_*x*_, *H*_*y*_, *H*_*z*_) in the center of each cluster is calculated and the relative numbers of clusters with components *H*_*x*_, *H*_*y*_, and *H*_*z*_ falling into each predefined interval *ΔH* are determined.

To obtain the partial probability densities of the second approximation, we generate clusters in the volume of the Lorentz sphere, the particle centers being randomly distributed. The magnetization directions of individual NPs are assigned in accordance with the probability density *P*_1_(*H*_*x*_,*H*_*y*_,*H*_*z*_) = *P*_1_(*H*_*x*_)*P*_1_(*H*_*y*_)*P*_1_(*H*_*z*_). Namely, the magnetic field ***H*** = (*H*_*x*_, *H*_*y*_, *H*_*z*_) acting on a specific nanoparticle of the cluster is set randomly, in accordance to *P*_1_(*H*_*x*_,*H*_*y*_,*H*_*z*_) values. Then, the average magnetization of this particle is determined by Eqs. (3) and (4). In this way, we can assign the magnetization of all ‘*N*_*p*_ − 1’ NPs of the cluster and calculate the total magnetic field acting on the test particle. If we repeat this procedure a sufficient number of times, we can determine the empirical probability density in the second approximation, *P*_2_(*H*_*x*_,*H*_*y*_,*H*_*z*_). These iterations are repeated until successively obtained probability densities, *P*_*i*_(*H*_*x*_,*H*_*y*_,*H*_*z*_), *i* = 1, 2, … converge to a certain limit. This limiting probability density is used in Eq. (5) to obtain the equilibrium magnetization of the assembly at a given *H*_0_ value.

To obtain the probability density *P*(*H*_*x*_,*H*_*y*_,*H*_*z*_) with an ~ 1% accuracy, it is enough to carry out only 3–4 iterations of this iterative procedure. The first iteration thus yields partial probability densities *P*_1_(*H*_*x*_), *P*_1_(*H*_*y*_) and *P*_1_(*H*_*z*_), which are very close to the Gaussian distribution, $P\left( H \right) = {{\exp \left( { - {{H^{2} } \mathord{\left/ {\vphantom {{H^{2} } {2\sigma^{2} }}} \right. \kern-\nulldelimiterspace} {2\sigma^{2} }}} \right)} \mathord{\left/ {\vphantom {{\exp \left( { - {{H^{2} } \mathord{\left/ {\vphantom {{H^{2} } {2\sigma^{2} }}} \right. \kern-\nulldelimiterspace} {2\sigma^{2} }}} \right)} {\sqrt {2\pi } \sigma }}} \right. \kern-\nulldelimiterspace} {\sqrt {2\pi } \sigma }}$, with some empirical standard deviations, $\sigma_{x}^{\left( 1 \right)}$, $\sigma_{y}^{\left( 1 \right)}$ and $\sigma_{z}^{\left( 1 \right)}$. As a result of the iterative procedure, we obtain series of standard deviations, $\sigma_{x}^{\left( i \right)}$, $\sigma_{y}^{\left( i \right)}$ and $\sigma_{z}^{\left( i \right)}$, *i* = 1, 2, … which quickly converge to some limiting values, $\sigma_{x}$, $\sigma_{y}$ and $\sigma_{z}$. Moreover, due to the axial symmetry of the problem an approximate equality $\sigma_{x}^{\left( i \right)}$ ≈ $\sigma_{y}^{\left( i \right)}$ is satisfied at each iteration step.

**Figure 7:**
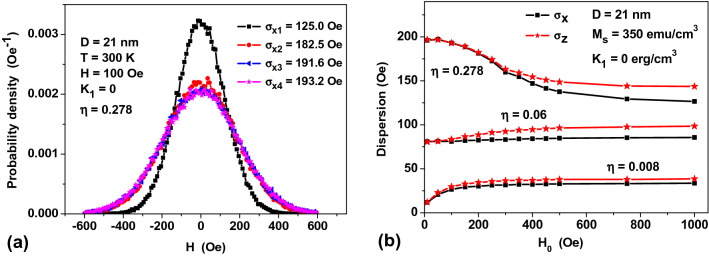
(**a**) Evolution of the empirical probability densities *P*^(*i*)^(*H*_*x*_) of the *H*_*x*_ component of random magnetic field for successive iterations *i* = 1–4; and (**b**) limiting empirical standard deviations of the probability densities of *H*_*x*_ and *H*_*z*_ random field components for assemblies of NPs with different *η* values over applied *H*_0_ field.

As an example, Fig. 7a shows the evolution of the empirical probability densities *P*_1_(*H*_*x*_)–*P*_4_(*H*_*x*_) for the *H*_*x*_ component of random magnetic field during four successive stages of the iterative procedure. To obtain empirical probability density, at each stage of the iterative procedure *N*_*exp*_ = 10^5^ numerical experiments were carried out in which spherical clusters consisting of *N*_*p*_ = 60 NPs of diameter *D* = 21 nm and cluster filling density *η* = 0.278 were created. To construct the empirical probability densities, the interval of magnetic fields (− 600, 600 Oe) was subdivided into 120 intervals each of 10 Oe. The particle centers inside the cluster volume were randomly distributed using the algorithm described in the “Methods” section. The particle magnetizations were assigned by means of the procedure described above and using Eqs. (3) and (4). As can be seen from Fig. 7a, the successively obtained *P*^(*i*)^(*H*_*x*_), *i* = 1–4, values can be described with a reasonable accuracy by the Gaussian distribution. The empirical standard deviations quickly converge to a constant limiting value. The empirical probability densities for the *H*_*y*_ and *H*_*z*_ components of the random magnetic field ***H*** are of the same form. As Fig. 7b shows, for small *H*_0_ values the limiting empirical standard deviations $\sigma_{x}$ and $\sigma_{z}$ turn out to be very close each other. As *H*_0_ increases, they bifurcate, but always $\sigma_{x}$ < $\sigma_{z}$. Moreover, $\sigma_{x}$ = $\sigma_{y}$ for the transverse components of the random magnetic field due to the axial symmetry of the problem.

**Figure 8:**
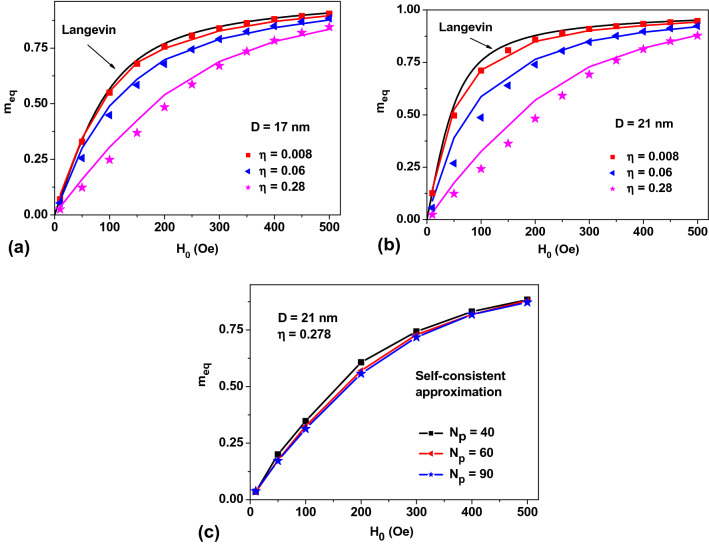
Comparison of *m*_*eq*_ values of an assembly of NPs with *K*_1_ = 0, *M*_*s*_ = 350 emu/cm^3^ calculated in the stochastic LL equation (solid lines), and self-consistent field approximation (dots) for NPs of (**a**) *D* = 17 nm and (**b**) *D* = 21 nm; and (**c**) of those obtained in the self-consistent approximation for different numbers *N*_p_ of NPs in the Lorentz sphere.

Figure 8a,b plot so obtained *m*_*eq*_ values over *H*_0_ for assemblies with *K*_1_ = 0. Solid lines represent the results of direct numerical calculation using the stochastic LL equation for NPs with diameters *D* = 17 and 21 nm, respectively, while the dots show the values calculated in the self-consistent approximation developed. The number of NPs in the Lorentz sphere in the latter case was fixed at *N*_*p*_ = 60, and only four cycles of the iteration procedure was carried out for every dot. Here, the maximum difference between the results of two calculations does not exceed 15%, which is due to the presence of the correlation effects. Obviously, the dynamics of the magnetic moments of closely located NPs should be strongly correlated, but this fact is not taken into account in the approximation developed. Figure 8c plots *m*_*eq*_ values of random assembly of NPs with *D* = 21 nm calculated for different numbers of NPs in the Lorentz sphere. An increase in the number of NPs in the Lorentz sphere in excess of *N*_*p*_ = 60 does not lead to any noticeable change in *m*_*eq*_ values.

As Fig. 7b shows, a difference between the self-consistent standard deviations $\sigma_{x}$ and $\sigma_{z}$ is usually small in a wide range of *H*_0_ ≤ 500 Oe. Assuming approximately $\sigma_{x} \approx \sigma_{z} = \sigma$ and performing calculations in a spherical coordinate system with the polar axis parallel to the direction of the applied *H*_0_ field, one can rewrite Eq. (5) as follows

7 $$\frac{{\left\langle {M_{z} } \right\rangle }}{{M_{s} }} = \sqrt {\frac{2}{\pi }} \int\limits_{0}^{\infty } {m_{L} \left( {\frac{{M_{s} VH}}{{k_{B} T}}} \right)} \exp \left( { - \frac{{H^{2} + H_{0}^{2} }}{{2\sigma^{2} }}} \right)\frac{{H^{2} \left( {\xi \cosh \xi - \sinh \xi } \right)}}{{\sigma^{3} \xi^{2} }}dH,$$

where *ξ* = *HH*_0_/*σ*^2^. In the limit *H*_0_ → 0 this integral is estimated to be

8 $$\frac{{\left\langle {M_{z} } \right\rangle }}{{M_{s} }} \approx \frac{2}{3}\sqrt {\frac{2}{\pi }} \frac{{m_{L} \left( {{{\sqrt 3 M_{s} V\sigma \left( 0 \right)} \mathord{\left/ {\vphantom {{\sqrt 3 M_{s} V\sigma \left( 0 \right)} {k_{B} T}}} \right. \kern-\nulldelimiterspace} {k_{B} T}}} \right)}}{\sigma \left( 0 \right)}H_{0} ,$$

where *σ*(0) is the standard deviation at *H*_0_ = 0. For characteristic values of the standard deviation, *σ*(0) ~ 100 Oe, the Langevin function *m*_*L*_ in Eq. (8) changes slowly. Thus, as Eq. (8) shows, with an increase in *σ*(0) value the initial magnetic susceptibility of the assembly decreases approximately as 1/*σ*(0). It can be shown that Eq. (8) accurately describes the initial linear portion of the *M*(*H*_0_) curves shown in Fig. 8, if one uses in Eq. (8) the corresponding *σ*(0) values obtained in a numerical simulation. Obviously, the decrease in the equilibrium assembly magnetization as a function of its density is due to a disorienting effect of the random magnetic field ***H***. Actually, under the influence of random magnetic field the magnetic moments of the NPs on average deviate from the *H*_0_ direction.

**Figure 9:**
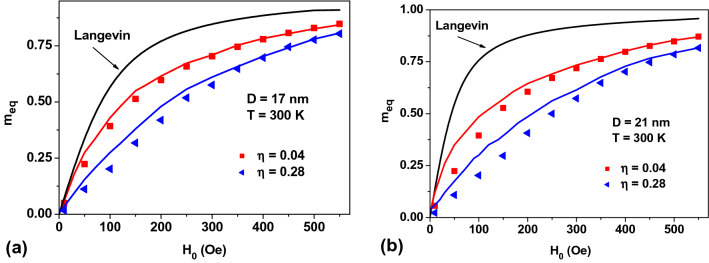
A comparison of m_eq_ values calculated for assemblies of random clusters of NPs by solving the stochastic LL equation (solid lines) and those obtained in the self-consistent field approximation (dots) for NPs of (**a**) *D* = 17 nm, and (**b**) *D* = 21 nm, with *K*_1_ = 10^5^ erg/cm^3^ and *M*_*s*_ = 350 emu/cm^3^.

Similar calculations of the equilibrium assembly magnetization in the self-consistent field approximation were also performed for random assemblies with *K*_1_ > 0 value. Instead of using Eqs. (3) and (4), in this case one has to assign the magnetizations of the NPs within the Lorentz sphere by means of the corresponding Gibbs principle taking into account the *K*_1_ value and the directions of easy anisotropy axes of various NPs in the formulas given in Ref.^32^. Figure 9a,b plot *m*_*eq*_ values over *H*_0_ for an assembly of NPs with *K*_1_ = 10^5^ erg/cm^3^, *M*_*s*_ = 350 emu/cm^3^ for the NPs of *D* = 17 and 21 nm, respectively. The magnetic field dependences of *m*_*eq*_ values obtained in the two different methods turn out to be sufficiently close in the entire 0–500 Oe range of the applied *H*_0_ fields.

For completeness, similar calculations of the equilibrium assembly magnetization have been carried out for dilute assemblies of elongated and oblate clusters of magnetic NPs with aspect ratios *D*_*z*_/*D* = 2.0 and *D*_*z*_/*D* = 0.5, where *D*_*z*_ and *D* are the longitudinal and transverse diameters of the spheroidal magnetic cluster, respectively. It is shown that for a given *H*_0_ value, the equilibrium assembly magnetization increases for an elongated cluster with an aspect ratio *D*_*z*_/*D* > 1 and decreases in the opposite case, *D*_*z*_/*D* < 1, in comparison with the results for a spherical cluster, *D*_*z*_/*D* = 1.0. These results are explained by the influence of the macroscopic demagnetizing field which acts inside the Lorentz sphere created in elongated or oblate spheroids.

## Conclusions

An assembly of single-domain magnetic NPs is a complex physical system whose properties are determined by many factors, such as the distribution of NPs in size and shape, the density of the assembly, and the value of the main magnetic parameters of the NPs. Behavior of the assembly depends also on the properties of the medium where the NPs are distributed, beside the applied magnetic field and the temperature. In contrast to classical plasma or quantum gases with Coulomb interaction^14–18^ magnetic particles interact via anisotropic magneto-dipole forces. Moreover, a SPMNP is characterized by an induced magnetization, which is nearly zero in the absence of magnetic field acting on the particle, contrary to elementary particles whose electric charge is fixed.

In this paper we study the properties of a SPM assembly of monodispersed NPs in a solid nonmagnetic matrix. The calculations performed take into account magnetic anisotropy and the magneto-dipole interaction of particles, but a contact exchange interaction is ignored between the NPs, as they are protected by thin nonmagnetic shells. This model differs significantly from that describing ferrofluids^46, 47,50,52,53^. In fluid NPs can rotate as a whole. In addition, they may redistribute to form chains of particles or dense conglomerates^46,47,50,52,53^.

To realize a SPM regime the sample temperature should be higher than the characteristic blocking temperature *T*_*b*_ of NPs. It is important that SPMNPs assembly relaxes to a thermodynamically equilibrium over a finite observation time. The fundamental physical quantity of a SPMNPs assembly is the equilibrium magnetization, *M*_*eq*_ = *M*_*eq*_(*H*_0_,*T*), which can be easily measured experimentally^23–29^. Theoretically, this value can be determined on the basis of the Gibbs principle^14–21^ as a derivative of the assembly’s free energy with respect to an applied field *H*_0_. However, a direct calculation of the Gibbs statistical integral for an assembly of interacting magnetic NPs involves great mathematical difficulties. In this paper, a new physically adequate method is used for calculating the *M*_*eq*_(*H*_0_,*T*) value of an assembly by solving the stochastic LL equation^56–60^. In contrast to the Monte-Carlo calculations^25,33–42,46,50^, the relaxation process to thermodynamic equilibrium in the assembly can be directly observed using the stochastic LL equation. Detailed calculations of the equilibrium magnetization were performed for dilute assemblies of magnetic clusters containing *N*_*p*_ = 60–100 NPs of a given diameter. The intensity of the magneto-dipole interaction inside the clusters can be controlled by changing the cluster filling density *η*.

In an assembly of weakly interacting NPs it is shown that due to the influence of magnetic anisotropy energy, equilibrium magnetization differs significantly from the Langevin law in the range of moderate and large *H*_0_ fields. Nevertheless, in sufficiently small *H*_0_ the *K*_1_ dependence of the equilibrium magnetization disappears. In this area the Langevin formula is valid and describes universal behavior of a dilute assembly. For the assemblies of iron oxide NPs studied here the universal behavior is observed over *H*_0_ ≤ 50 Oe. However, for dense assemblies with a noticeable influence of the magneto-dipole interaction a significant dependence of the initial susceptibility on the density is revealed. A difference of the initial susceptibility over the Langevin value serves as a validity of the influence of the magneto-dipole interaction on the assembly properties.

In this paper a new approach to describe the influence of random magnetic field acting on NPs in a dense assembly is proposed. In effective field theories^46–50,52^ it is assumed that a typical nanoparticle of the assembly is subjected to some self-consistent magnetic field, which takes into account the influence of the magnetic fields of the surrounding NPs. However, in a real assembly each nanoparticle is under the influence of its own local magnetic field which contains a random component. In this paper the probability densities of the components of random magnetic field acting on a typical magnetic nanoparticle are calculated. The self-consistent probability densities of random field components are described by Gaussian distribution. Thus, the standard deviation in the Gaussian distribution becomes an important parameter of the theory. Knowing the probability density of the components of random magnetic field it is possible to calculate the equilibrium magnetization of the assembly in the given approximation as a function of applied *H*_0_ field. It is shown that the approach developed satisfactorily describes the numerical results obtained for the equilibrium *M*(*H*_0_) curve with the help of stochastic LL equation.

The effect of intense magneto-dipole interaction on the properties of an assembly of magnetic NPs is usually explained^25^ either by a change in the characteristic height of energy barriers between potential wells of magnetic NPs, or by some collective processes that simultaneously affect the magnetic state of closely spaced magnetic NPs. Based on Eqs. (5) and (6) in this work it is shown that a decrease in the equilibrium magnetization of an interacting assembly as a function of its density can be explained by the disorienting effect of random magnetic field. This leads, on average, to a deviation of the magnetic moments of the NPs from the applied magnetic field direction. In this connection, it is worth noting that the broadening of spectral lines in a high temperature plasma was successfully explained by the action of a random electric microfield, the statistical properties of which are described by Holtsmark^62^ or similar^63^ distributions.

## Methods

### Stochastic Landau–Lifshitz equation

Dynamics of a unit magnetization vector $\vec{\alpha }_{i}$ of a single-domain nanoparticle *i* of the cluster is determined by the stochastic LL equation^56–60^

9 $$\frac{{\partial \vec{\alpha }_{i} }}{\partial t} = - \gamma_{1} \vec{\alpha }_{i} \times \left( {\vec{H}_{ef,i} + \vec{H}_{th,i} } \right) - \kappa \gamma_{1} \vec{\alpha }_{i} \times \left( {\vec{\alpha }_{i} \times \left( {\vec{H}_{ef,i} + \vec{H}_{th,i} } \right)} \right),$$

where *γ* is the gyromagnetic ratio, *κ* is phenomenological damping constant, *γ*_1_ = *γ*/(1 + *κ*^2^), $\vec{H}_{ef,i}$ is the effective magnetic field and $\vec{H}_{th,i}$ is the thermal field. The effective magnetic field acting on a separate nanoparticle can be calculated as a derivative of the total cluster energy

10 $$\vec{H}_{ef,i} = - \frac{\partial W}{{VM_{s} \partial \vec{\alpha }_{i} }}.$$

The total magnetic energy of the cluster *W* = *W*_*a*_ + *W*_*Z*_ + *W*_*m*_ is a sum of the magneto-crystalline anisotropy energy *W*_*a*_, Zeeman energy *W*_*Z*_ of the particles in applied magnetic field $\vec{H}_{0}$, and the energy *W*_*m*_ of mutual magneto-dipole interaction of NPs in the cluster.

For spherical NPs with uniaxial type of magnetic anisotropy the magneto-crystalline anisotropy energy is given by

11 $$W_{a} = K_{1} V\sum\limits_{i = 1}^{{N_{p} }} {\left[ {1 - \left( {\vec{\alpha }_{i} \vec{e}_{i} } \right)^{2} } \right]} ,$$

where ***e***_*i*_ is the orientation of the easy anisotropy axis of *i*-th particle of the cluster. Zeeman energy *W*_*Z*_ of the cluster in an applied magnetic field *H*_0_ is given by

12 $$W_{Z} = - M_{s} V\sum\limits_{i = 1}^{{N_{p} }} {\vec{\alpha }_{i} \vec{H}_{0} } .$$

Next, for spherical uniformly magnetized NPs the magnetostatic energy of the cluster can be represented as the energy of the point interacting dipoles located at the particle centers ***r***_*i*_ within the cluster. Then the magneto-dipole interacting energy is

13 $$W_{m} = \frac{{M_{s}^{2} V^{2} }}{2}\sum\limits_{i \ne j} {\frac{{\vec{\alpha }_{i} \vec{\alpha }_{j} - 3\left( {\vec{\alpha }_{i} \vec{n}_{ij} } \right)\left( {\vec{\alpha }_{j} \vec{n}_{ij} } \right)}}{{\left| {\vec{r}_{i} - \vec{r}_{j} } \right|^{3} }}} ,$$

where ***n***_*ij*_ is a unit vector along the centers of *i*-th and *j*-th particles, respectively.

Thus, the effective magnetic field acting on the *i*-th nanoparticle of the cluster is given by

14 $$\vec{H}_{ef,i} = H_{a} \left( {\vec{\alpha }_{i} \vec{e}_{i} } \right)\vec{e}_{i} + \vec{H}_{0} - M_{s} V\sum\limits_{j \ne i} {\frac{{\vec{\alpha }_{j} - 3\left( {\vec{\alpha }_{j} \vec{n}_{ij} } \right)\vec{n}_{ij} }}{{\left| {\vec{r}_{i} - \vec{r}_{j} } \right|^{3} }}} .$$

The thermal fields, $\vec{H}_{th,i}$, *i* = 1, 2, ... *N*_*p*_, acting on various NPs of the cluster are statistically independent, with the following statistical properties^56^ of their components for every nanoparticle

15 $$\left\langle {H_{th}^{(\alpha )} \left( t \right)} \right\rangle = 0,\quad \left\langle {H_{th}^{(\alpha )} \left( t \right)H_{th}^{(\beta )} \left( {t_{1} } \right)} \right\rangle = \frac{{2k_{B} T\kappa }}{{\gamma M_{s} V}}\delta_{\alpha \beta } \delta \left( {t - t_{1} } \right),\quad \alpha ,\beta = \left( {x,y,z} \right).$$

here *δ*_*αβ*_ is the Kroneker symbol, and *δ*(*t*) is the delta function.

The procedure for solving these equations is described in detail in Refs.^57–59^.

### Random 3D clusters of NPs

In the Monte-Carlo calculations performed to study the SPMNPs assemblies the nanoparticle positions were randomly generated^36,40^ on nodes of simple cubic lattices with a certain lattice parameter. This numerical algorithm can hardly be considered as truly random. In particular, it completely prevents the appearance of numerous assembly configurations where certain NPs turn out to be very close to each other, i.e. closer than the lattice parameter chosen. In the present study the 3D clusters consisting of *N*_*p*_ identical magnetic NPs with truly random positions were created using numerical algorithm developed in Ref.^12^. First, a dense and approximately uniform set of *N* random points {***ρ***_*i*_} was created in a sphere of the radius *R*_*cl*_, so that |***ρ***_*i*_| ≤ *R*_*cl*_, *i* = 1, 2, ... *N*, for *N* ≫ *N*_*p*_. The first random point ***ρ***_1_ can be chosen as a center of the first nanoparticle of the assembly, ***r***_1_ = ***ρ***_1_. Then it is necessary to remove all points with coordinates |***ρ***_*i*_* − r*_1_| ≤ *D* from the initial set of the random points. Any random point in the remaining set of points can serve as a center of second nanoparticle of the assembly, for example, ***r***_2_ = ***ρ***_2_. Continuing this procedure, one can assign centers to all *N*_*p*_ NPs within the cluster volume. Moreover, none of the NPs of the assembly will be in a direct contact with the surrounding particles. This algorithm allows to create random 3D clusters of magnetic NPs with filling densities *η* < 0.5. The orientations of the easy anisotropy axes {***e***_*i*_}, *i* = 1, 2, … *N*_*p*_, of NPs in a random 3D clusters are chosen randomly in a sphere.

## Data Availability

No data-sets were generated or analyzed during the current study.
